# Non-Viral Delivery Systems to Transport Nucleic Acids for Inherited Retinal Disorders

**DOI:** 10.3390/ph18010087

**Published:** 2025-01-13

**Authors:** Md Jobair Jony, Ameya Joshi, Alekha Dash, Surabhi Shukla

**Affiliations:** Department of Pharmacy Sciences, School of Pharmacy and Health Professions, Creighton University, Omaha, NE 68178, USA; mdjobairhossenjony@creighton.edu (M.J.J.); ameyajoshi@creighton.edu (A.J.); alekhadash@creighton.edu (A.D.)

**Keywords:** non-viral delivery system, liposome, gene therapies, inherited retinal disorders, viral based vector, micelles, dendrimers

## Abstract

Inherited retinal disorders (IRDs) represent a group of challenging genetic conditions that often lead to severe visual impairment or blindness. The complexity of these disorders, arising from their diverse genetic causes and the unique structural and functional aspects of retinal cells, has made developing effective treatments particularly challenging. Recent advancements in gene therapy, especially non-viral nucleic acid delivery systems like liposomes, solid lipid nanoparticles, dendrimers, and polymersomes, offer promising solutions. These systems provide advantages over viral vectors, including reduced immunogenicity and enhanced targeting capabilities. This review delves into introduction of common IRDs such as Leber congenital amaurosis, retinitis pigmentosa, Usher syndrome, macular dystrophies, and choroideremia and critically assesses current treatments including neuroprotective agents, cellular therapy, and gene therapy along with their limitations. The focus is on the emerging role of non-viral delivery systems, which promise to address the current limitations of specificity, untoward effects, and immunogenicity in existing gene therapies. Additionally, this review covers recent clinical trial developments in gene therapy for retinal disorders.

## 1. Introduction

The eye is a complex organ with unique anatomical and immune-privileged characteristics, making it an attractive target for gene therapy. This treatment strategy involves inserting genetic material into cells to correct abnormal genes or to produce therapeutic proteins. Among various genetic conditions that affect the eye, inherited retinal disorders (IRDs) stand out due to their heterogenicity and complexity. IRDs encompass a range of genetic eye diseases, including retinitis pigmentosa, Leber congenital amaurosis, macular dystrophies, choroideremia, and Stargardt disease, which typically result in progressive vision loss or blindness [1]. These disorders affect approximately 1 in 2000 individuals globally, and with limited treatment options, there is a critical need for innovative treatment approaches [2]. Gene therapy for eye diseases presents a prospective approach to treat IRDs at their root cause. The ideal gene therapy method would be one that can efficiently deliver genes to specific target cells without causing toxicity or immune responses, enable regulated expression of the therapeutic gene and avoid integration-related mutagenic risks [3,4]. It should also be practical for clinical use and scalable in production.

While viral vectors such as adeno-associated viruses (AAV), recombinant adeno-associated viruses (rAAV), and lentiviruses have been prominently used in ocular gene therapy, they come with potential risks like rapid clearance from the circulation, the reduced capacity to carry a large amount of genetic information, viremic infectivity, and immunogenicity [5,6]. As a result, non-viral vectors have gained interest as a safer alternative. These vectors, less likely to elicit immune responses in mammalian cells, offer a safer profile with low toxicity [7]. They are also more manageable in handling and production, although they tend to have lower transfection efficiency [8].

In this review, we aim to discuss the use for non-viral vectors in ocular gene delivery, highlighting both the challenges and advancements in this rapidly evolving area. Special attention is given to the exploration of nanoparticle-based delivery systems, which show promise in treating inherited retinal disorders. We also mention the current state of clinical trials, shedding light on the cutting-edge developments in this domain.

## 2. Eye Anatomy and Physiology

**Figure 1:**
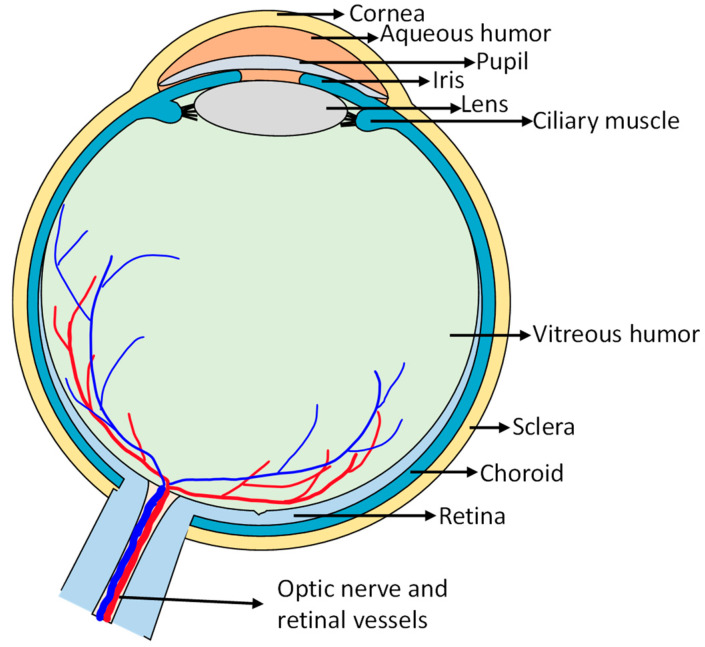
The anatomy of the eye.

**Figure 2:**
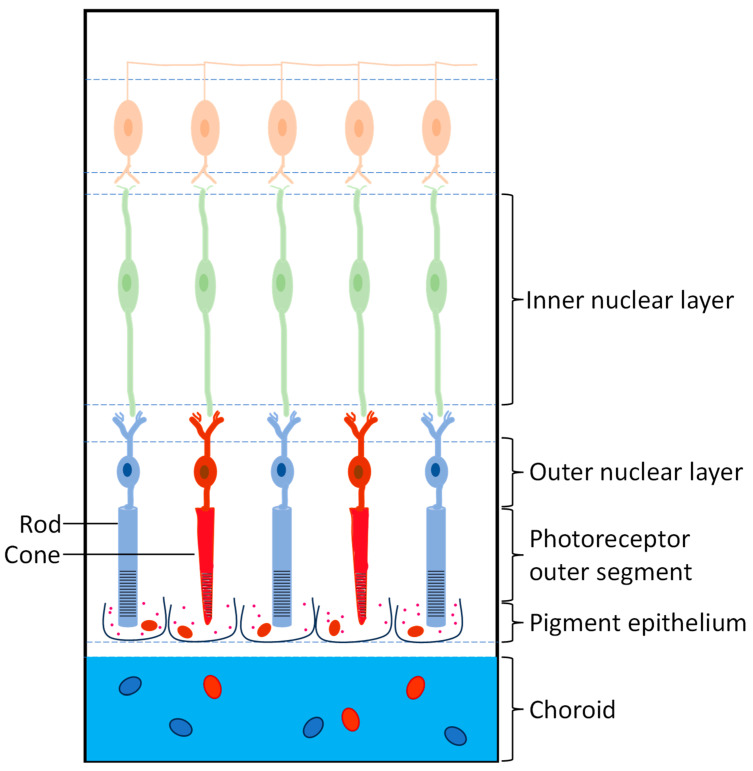
The structure of the retina and its major cell types.

The human eye is a complex and delicate organ that plays a critical role in our ability to see and interpret the world around us. Its different parts work in tandem to gather and process visual information. It can be compartmentalized into two distinct segments, i.e., anterior segment and posterior segment [9]. The anterior segment consists of the cornea, conjunctiva, aqueous humor, iris, ciliary body, and lens. The posterior segment is composed of the sclera, choroid, Bruch’s membrane, retinal pigment epithelium (RPE), neural retina, and vitreous humor (Figure 1) [10]. The retina is a thin layer of tissue that lines the inner surface of the eye and functions for detecting light and converting it into electrical impulses that are transmitted to the brain [11]. It contains specialized cells called photoreceptors, which include rods and cones (Figure 2). Rods are responsible for detecting light in dim conditions and concentrate in the peripheral part of the retina. Cones control color vision and are densely packed in the central part of the retina, known as the macula, which is a small, yellow spot at the center of the retina, and it provides the sharpest and clearest vision. It contains a high number of cones and is responsible for fine detail and color vision [10].

The only type of rod found in the mammalian retina is rhodopsin. In contrast, there are two main types of cones—S cone opsin, also known as blue-sensitive opsin, and M cone opsin, also known as green-sensitive opsin—are the two forms of cone opsin found in most mammals, including mice. In humans and primates, there is an additional L cone opsin (also known as red-sensitive opsin) that is sensitive to long wavelengths (red). Together, these three cone opsins allow for trichromatic vision [12].

RPE cells are responsible for various critical functions, including phagocytosis of toxic products, reconstitution, maintenance of the blood–retinal barrier, reduction in phototoxic damage to the retina, nourishing of the cones and rods, fixation of the retina, and metabolism of retinoids [10,13,14].

The retina’s structural and functional integrity depend on the proper functioning of its various components, and any disruptions to their delicate balance can lead to retinal disorders and vision loss.

## 3. Challenges in Ocular Gene Delivery

The depletion of a drug after its administration in the eye poses a significant hurdle in ocular treatments. This loss can occur through various pathways including the ocular surface barrier, secretion and outward flushing of lacrimal fluid, and the blood–ocular barrier [15]. This makes it difficult to achieve effective therapeutic concentrations in the eye, challenging the efficacy of ocular medications.

### 3.1. Ocular Surface Drug Depletion

Even though the lacrimal turnover rate is only about 1 μL/min, the excess volume of the instilled fluid is flown to the nasolacrimal duct rapidly in a couple of minutes [16,17]. Another factor contributing to inefficient drug delivery is the systemic rather than ocular absorption of the drug. This absorption can occur through local blood capillaries in the conjunctival sac or after the drug solution reaches the nasal cavity [16,18,19]. In most cases, a significant portion of the drug with a small molecular weight is quickly absorbed into the systemic circulation within a few minutes. This rapid systemic absorption is in stark contrast to the notably low ocular bioavailability, which is less than 5% [16,17].

### 3.2. Lacrimal Fluid Barriers

The corneal epithelium, forming a barrier upon the maturation of epithelial cells, significantly limits drug absorption from the lacrimal fluid into the eye [16,20]. As these cells migrate from the limbal region to the cornea’s center, they create tight junctions at the apical surface, impeding paracellular drug permeation [16,21]. This structure results in notably higher permeability for lipophilic drugs compared to hydrophilic ones [22]. Conversely, the conjunctiva, with a larger surface area and a more permeable structure, offers a potential absorption route for hydrophilic and larger molecules like proteins and peptides. This difference in permeability is crucial in ocular drug delivery, as most clinically used drugs are small and lipophilic, favoring the corneal route [16,23].

### 3.3. Blood–Ocular Barriers

The blood–ocular barriers, crucial in protecting the eye from xenobiotics, consist of the blood–aqueous barrier (BAB) and the blood–retina barrier (BRB). The BAB, part of the anterior blood–eye barrier, comprises endothelial cells in the uvea, limiting plasma albumin and hydrophilic drug access to the aqueous humor. Inflammation can disrupt this barrier, leading to unrestricted drug distribution in the anterior chamber [16]. The posterior barrier, BRB, involves retinal pigment epithelium (RPE) and retinal capillaries, regulating drug entry from the blood to the posterior chamber [15,24,25]. The choroid’s vasculature, despite its high blood flow, allows for limited drug access due to its connection with the retina through the RPE and retinal endothelia. The tight junctions in the endothelial cells of the retina and RPE restrict drug molecules’ entry into intraocular spaces. The permeability of these barriers varies, with the RPE allowing for more predictable drug passage compared to the vascular component of the BRB [16]. Particle size is critical in drug permeation, with retinal capillaries blocking larger particles but allowing for small molecules. The choroid facilitates drug distribution in blood and extravascular spaces but restricts access to the retina [15]. Techniques like PEGylated liposomes have been developed to enhance drug delivery across these barriers, with particle size and surface charge being key factors in effective retinal permeation [15,26].

## 4. Common Inherited Retinal Disorders

Inherited retinal degenerations (IRDs) encompass a range of progressive conditions that often lead to blindness [1]. These diseases are marked by genetic mutations critical to retinal health, resulting in the gradual loss of photoreceptor cells and irreversible vision impairment. Despite the diverse clinical manifestations and over 260 identified disease-causing genes, IRDs share a common trajectory towards photoreceptor death and consequent vision loss [27].

### 4.1. Leber Congenital Amaurosis

Leber congenital amaurosis (LCA), is a hereditary retinal dystrophy. This disorder is characterized by significant vision loss from birth or early infancy, wandering nystagmus, amaurotic pupils, and a pigmented retina [28].

LCA’s complexity arises from its genetic heterogeneity, with mutations in various genes linked to multiple visual pathways. One subtype of LCA, related to the retinal pigment epithelium 65 (RPE65) gene, is known as LCA2. In patients with this condition, there is a notable loss of photoreceptor function leading to a profound impact on vision. The condition’s inherent heterogeneity poses significant challenges in understanding and categorizing the disease, due to the varying genetic factors involved [1]. Patients with mutations in this gene exhibit severe photoreceptor dysfunction and degeneration, with markedly reduced or absent electroretinogram (ERG) responses from birth or initial presentation [29].

LCA1 is a genetic disorder resulting from homozygous or compound heterozygous mutations in the GUCY2D gene, which is responsible for encoding retinal guanylyl cyclase 1. This enzyme is mainly found in the outer segment of cone cells in the retina but is also present in rod cells [30]. Mutations in GUCY2D lead to significantly reduced visual acuity, nystagmus, and pronounced dysfunction of the photoreceptors, as observed in ERG tests [1].

### 4.2. Retinitis Pigmentosa

Retinitis pigmentosa (RP), an inherited retinopathy, affects more than 1.5 million people. Among all forms of inherited retinal dystrophies (IRD), it is one the most prevalent worldwide [31,32]. Many instances are brought on by mutations in only one particular gene—RPE65, which is a substantial cause of blindness [10]. As is apparent from its name, the underlying pathology includes degeneration of photoreceptor cells, i.e., rods and cones, and deposition of retinal pigments, giving the retina a pigmented appearance [33]. The condition can manifest at any age, from childhood to adulthood. Typically, it starts in the rod photoreceptors and advances to the progressive degeneration of the cones. As a result, the initial manifestation is nyctalopia (night blindness), which leads to a gradual narrowing of the visual field before central vision loss and complete blindness [10]. The mutations and genetics that cause RP are quite intricate as well as heterogeneous. It has also been associated with gene mutations linked to other inherited retinopathies. Presently, more than 40 genes have been found to be connected to RP [34]. Non-syndromic RP has been associated with over 3000 mutations in over 50 unique genes or loci [35]. The genetic trait of RP can be inherited through different modes of inheritance, including X-linked (5–15%), autosomal dominant (15–25%), autosomal recessive (5–20%), and unknown patterns (40–50%) [36]. The major responsible genes are RHO, IMPDH1, RPRF, RP1, PRPH2, GUCA1B, PRPF8, SAG, SEMA4AKLHL7, NR2E3, and SNRNP200 for ADRP; ABCA4, USH2A, RPE65, EYS, CERKL, CRB1, PDE6A, SAG, and PDE6B for ARRP; and RPGR and RP1 for XLRP [32,36,37].

### 4.3. Usher Syndrome

Usher syndrome is a genetic disorder that is autosomal recessive, marked by retinitis pigmentosa, a gradual loss of hearing, and possible issues with vestibular function [38]. Moreover, there are over fifteen identified loci linked to Usher syndrome, impacting both the photoreceptors and elements of the inner ear like the hair bundle and synapse [39]. This syndrome is classified into three primary categories, USH1, USH2, and USH3, differentiated by clinical symptoms. Individuals with USH1 typically have severe hearing loss from birth, problems with vestibular function, and early-onset retinitis pigmentosa (RP). Those with USH2 usually experience moderate hearing loss and normal vestibular function, developing RP in early adulthood. USH3 is characterized by progressive hearing loss, occasional vestibular dysfunction, and varied onset of RP [40].

### 4.4. Macular Dystrophies

Stargardt disease is recognized as the most prevalent form of macular dystrophy [41]. It is characterized by the widespread deposition of lipofuscin (bisretinoids) in the retinal pigment epithelium (RPE), which gives rise to the classical fundus appearance of retinal flecks. The spectrum of disease is highly variable, in terms of the age of onset, clinical features, rate of progression and extent of retinal involvement, ranging from isolated macular disease to generalized cone and rod system involvement [42,43,44,45,46,47,48].

Best disease (BD), the second most common macular dystrophy, is attributed to dominant mutations in the BEST1 gene [48,49]. BD often coincides with hypermetropia, necessitating early correction to lower amblyopia risk. ARB, linked with more severe hypermetropia, also poses a high risk of angle-closure glaucoma, requiring preventive measures. BD’s typical feature is a bilateral, symmetrical egg yolk-like lesion at the fovea. Initially, the fundus may appear normal or show minimal changes, but the lesion can evolve, leading to a “pseudohypopyon” stage with subretinal material settling at the bottom. Early stages show normal vision, but vision reduction begins from the third stage, progressing to more advanced stages marked by further material breakdown and eventual atrophy [41,48].

### 4.5. Choroideremia

Choroideremia (CHM) is estimated to affect approximately 1 in 50,000 male patients [50]. It is an X-linked inherited retinal disease (IRD) marked by the degeneration of the retina, the retinal pigment epithelium (RPE), and the choroid caused by deletion or mutation of the CHM gene, encoding Rab escort protein-1 (REP1) [51,52]. The protein is vital for the health of the choroid, RPE, and neurosensory retina. Rab proteins, essential for cellular functions and particularly active in the eyes, are involved in the transport of proteins necessary for intracellular signaling in photoreceptors and the phagocytosis and breakdown of outer segment disc membranes in RPE cells. The disease manifests as choroid atrophy, resulting in a pale fundus due to the illumination of the sclera behind the degenerating choroid. Clinically, choroideremia typically begins with night blindness and progressively leads to a decrease in peripheral vision [1,53].

## 5. Current Therapeutic Approaches for Inherited Retinal Disorders

At present, there are several approaches to manage inherited retinal disorders (IRDs). The significant heterogeneity of these diseases impedes the progress of a universal remedy for many patients. In all these conditions, treatment ought to be commenced at the earliest to reduce the extent of irreversible damage to the retina.

### 5.1. Neuroprotective Agents

Recent developments in neuroprotective agents for inherited retinal diseases (IRDs) have shown significant promise in preserving neuronal structure and function, thus slowing vision loss. These neuroprotective strategies encompass a wide range of therapeutics, including neuropeptides, exosomes, mitochondrial-derived peptides, complement inhibitors, senolytics, autophagy enhancers, and antioxidants [54].

A variety of novel therapeutic modalities are being examined for photoreceptor neuroprotection, particularly in conditions such as age-related macular degeneration, inherited retinal dystrophies, and macular telangiectasia type 2. These advancements highlight the evolving landscape of treatment options aimed at mitigating the progression of these debilitating diseases [55]. Additionally, the neuroprotective activity of pigment epithelium-derived factor is being explored, with a focus on targeting specific molecular pathways. This approach emphasizes the need for deep analysis and characterization of specific targets for neuroprotection, offering new perspectives in the treatment of inherited retinal degeneration [56].

Moreover, numerous investigational neuroprotective compounds are currently in clinical trials, underscoring the ongoing efforts to identify effective treatments for retinal diseases. These trials are crucial in validating the efficacy and safety of these neuroprotective agents, paving the way for potential future therapies [57].

Promising research investigates interventions to promote cone survival and function in animal models of retinitis pigmentosa, potentially preventing secondary cone degeneration [58].

Other studies have explored treatments like D-cis-diltiazem for rescuing photoreceptors in specific retinal diseases, with varying results among different animal models [59,60].

### 5.2. Cellular Therapy

Cellular therapy for inherited retinal diseases (IRDs) represents a significant advancement in ophthalmology. Cell replacement therapy is one such innovative approach, aiming to replace dead or damaged retinal cells using a variety of cell sources. This includes mesenchymal stem cells, peripheral or fetal retinal pigment epithelium cells, human embryonic stem cells (hESCs), and human-induced pluripotent stem cells (hiPSCs) [61].

Cell-based therapies are being explored for a range of IRDs, such as Leber congenital amaurosis, choroideremia, retinitis pigmentosa, Usher syndrome, X-linked retinoschisis, Leber hereditary optic neuropathy, and achromatopsia. These approaches are currently being updated and examined through randomized control trials.

Stem cell therapy has emerged as a promising therapeutic approach for IRDs, including retinitis pigmentosa and Stargardt disease (STGD). This involves the introduction of stem cells that can replace degenerated cells, delivered to target tissues like the photoreceptors and RPE through various systems such as subretinal, intravitreal, or suprachoroidal delivery [61,62].

These therapeutic strategies offer hope for patients with IRDs. As research continues to evolve, these innovative treatments hold the potential to transform the management and prognosis of IRDs significantly.

### 5.3. Gene Therapy

Gene therapy is the delivery of targeted nucleotide fragments to upregulate/downregulate expression in a tissue, to treat the underlying etiology and prevent/cure/mitigate a disease condition [63]. With regards to Inherited retinal disorders (IRDs), gene therapy can be promising in that it can be used to downregulate degenerative processes. However, IRDs pose challenges in genetic medicine due to their complexity. Gene therapy offers potential treatments, but its gene specificity necessitates extensive development, including animal studies, clinical trials, and regulatory approvals. This targeted approach is exemplified by Luxturna, an RPE-65 gene therapy product, which is a significant financial investment at approximately USD 450,000 per injection in the United States [61].

Recent advancements in genetic characterization have identified over 260 causative mutations linked to IRDs. Previously considered incurable, gene supplementation therapy now offers significant hope. This approach involves replacing a disease-causing gene with a functional copy, aiming to restore or preserve vision [4].

Gene therapy’s efficacy is further enhanced by advances in DNA delivery systems and improved genetic diagnostics for IRDs. The monogenic nature of most IRDs makes the retina an ideal target for gene therapy, as it allows for direct delivery of genetic vectors to the affected area [64].

For autosomal dominant IRDs, alternative strategies are being explored. Techniques like CRISPR/Cas9 or antisense oligonucleotides are employed to edit or deplete the mutant allele or gene product. This area of retinal gene therapy research is rapidly evolving, with various promising approaches in the preclinical and clinical development stages [65].

## 6. Nucleic Acids for Inherited Retinal Diseases

### 6.1. DNA Therapies

#### 6.1.1. Gene Augmentation

Gene augmentation therapy, also known as gene replacement therapy, involves introducing a normal gene copy into host cells. Adeno-associated viruses (AAVs) are the primary vectors to carry a specific gene for inherited retinal diseases (IRDs) due to their retinal cell tropism and low immunogenicity [66]. Lentiviruses and nanoparticles are also being studied for their larger cargo capacities [67]. This strategy may not suit in dominant conditions requiring mutated allele inactivation [68,69,70].

#### 6.1.2. Genome Editing

In recent years, genome editing has gained significant attention as an alternative to gene augmentation. Zinc finger nucleases (ZFNs) and transcription activator-like effector nucleases (TALENs) are early genome editing tools that induce genetic modifications via double-strand breaks (DSBs) that activate DNA repair pathways [71,72]. ZFNs demonstrated potential in treating IRD by increasing homologous recombination in human embryonic cells with an RHO mutation and TALENs improved retinal function in mice by correcting the Crb1rd8 allele. A disadvantage of ZFN as genome editing tool is its lack of simplicity due to difficulty of assembling a zinc finger domain to bind an extended stretch of nucleotide sequence with high affinity. It has limited target selection sites for open-source ZFN of target binding sites, about 1 in every 200 bps in a random DNA sequence. Another significant issue with the use of ZFN as a genomic editing tool is that it creates double-stranded breaks not only at the desired target site but also at off-target sites. Like ZFN, off-target effects are a considerable issue with TALENs. The large size of TALENs, approximately 3 kb, makes them unsuitable for therapeutic application where it needs to be delivered by AAV, which has a limited cargo capacity of 5 kb [73,74].

The CRISPR/Cas system, particularly CRISPR/Cas9, is more advanced, allowing for simultaneous editing of multiple genes. CRISPAR/Cas9 has advantage of simplicity of target design since it is dependent on ribonucleotide complex formation rather than protein–DNA recognition. The designing of guide RNAs to target any sequence in the genome is a very economical and easy process. The efficiency of introducing modification is excellent, as it can be performed easily by direct injection of RNA encoding the Cas protein and guide the RNA into developing mouse embryos as compared to the conventional homologous recombination, which has low efficiency in mammalian cells and model organisms. This also has advantage over classical homologous recombination techniques, as it reduces the cumbersome process of transfection and selection of mouse embryonic stem cells, which are necessary for creating targeted mutant mice. CRISPR/Cas9 can be directly delivered to human cells along with plasmid expressing Cas9 endonuclease and required crRNA components. Additionally, a CRISPAR/Cas-mediated genome editing tool has been successfully used in several other models such as zebrafish and bacteria [75].

### 6.2. RNA Therapies

#### 6.2.1. Splicing Modulation

Antisense oligonucleotides (AONs) play a crucial role in treating IRDs caused by mutations affecting splicing, which account for about 15% of all cases [76,77]. They have evolved from simple antisense RNA molecules to chemically modified forms to resist nuclease degradation, leading to various generations of AONs [77,78]. The first AON-based drug, Fomivirsen (Vitravene), was used to treat cytomegalovirus retinitis in immunocompromised patients [79,80].

Trans-splicing provides an innovative way to correct mRNA mutations. This method has shown success in correcting mutations in RHO and CEP290 genes in IRDs [70].

U1 spliceosomal RNA, vital for recognizing exonic splice donor sites, has led to modified U1 snRNA’s use as a therapeutic option, particularly effective in correcting certain mutations in vitro [70,81,82].

#### 6.2.2. Post-Transcriptional Gene Silencing

Both hammerhead ribozymes (hhRzs) and short interference RNA (siRNA) facilitate the targeted cleavage of specific mRNA sequences. Interference RNA (iRNA) molecules inhibit gene expression by binding to specific mRNAs [70]. siRNA has shown promising results in age-related macular degeneration treatment [83].

Hammerhead ribozymes (hhRzs) are small RNA molecules that enzymatically cleave polyribonucleotides. They consist of three helices and a catalytically active core, enabling them to target and cleave specific mRNA. hhRz has been used effectively against incorrect RHO transcripts in dominant retinitis pigmentosa, a common IRD subtype [84].

RNAse H-dependent antisense oligonucleotides (AONs) degrade transcripts in a specific and sometimes allele-specific manner [70]. Their ability to activate RNAse H1 allows them to cleave RNA within DNA/RNA hybrids [85]. AONs have shown effectiveness in rodent models with RHO mutations, preserving photoreceptor function, and have been explored for NR2E3 variant-related autosomal dominant RP [85].

## 7. Limitations of Current Therapies

Inherited retinal disorders (IRDs), a group of diseases caused by genetic mutations affecting proteins in the retina, significantly influencing their study, work, and life patterns [86]. Despite significant advances in gene therapy, the field of inherited retinal disease treatment faces several inherent limitations.

The search for effective neuroprotective therapies for retinal diseases is ongoing. While a range of novel therapeutic modalities, including agents targeting complement pathways, stem cells, gene therapies, and neurotrophic factors, have been explored in clinical trials, their clinical utility remains limited [55]. The transition of these therapies from preclinical to clinical realms has been particularly challenging due to issues with animal models, reproducibility of preclinical data, and meaningful clinical trial endpoints. Addressing these issues, like developing better animal models, defining guidelines for preclinical evaluation, and identifying biomarkers as surrogate endpoints, is essential [87].

Cellular therapy, especially stem cell therapy, for retinal diseases like retinitis pigmentosa and Stargardt’s macular dystrophy, has shown potential yet faces significant limitations. Though early-phase studies have demonstrated safety, significant efficacy has not been uniformly reported. Challenges include optimizing cell sources, immunosuppression regimens, surgical approaches, and outcome measures. The lack of FDA-approved treatments and the rise of unregulated “cell therapy” clinics, which have led to severe complications and vision loss in some cases, further complicate the landscape [62,88]. These factors highlight the need for more robust, large-scale clinical trials and regulatory oversight.

Gene therapy for inherited eye disorders like Leber congenital amaurosis (LCA) initially improves vision by introducing normal genes into the retina through a harmless virus. Although the therapy initially enhances light sensitivity and vision, the area of improvement reduces within a few years, as the therapy does not prevent photoreceptor cell death. Thus, while it offers significant benefits for incurable conditions, its effects are not permanent [89]. Moreover, there are other challenges, such as identifying the specific genes responsible for these conditions and managing gain-of-function mutations that complicate treatment. Furthermore, the selection of vectors is another challenge. Viral vectors, while offering higher transfection efficiency compared to non-viral vectors, encounter a range of limitations that impact their in vivo applications [90]. These challenges include rapid clearance from the bloodstream, a constrained capacity for carrying large quantities of genetic material, and the potential risks of toxicity and immunogenicity. Such drawbacks also limit the possibility of their repeated administration [6]. Effective targeting of therapy to affected retinal cells and avoiding retinal toxicity are crucial for successful outcomes [91].

While current therapies offer potential for treating IRDs, they are currently limited by challenges in clinical translation, long-term efficacy, and safety concerns.

## 8. Nanostructures Used in Nucleic Acid Delivery

**Figure 3:**
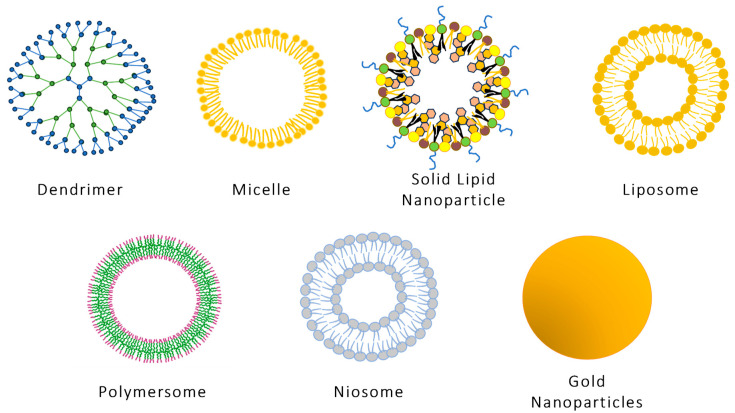
Schematic illustration of different nanotechnology-based ocular delivery systems.

Non-viral gene therapy delivery systems are emerging as safer substitutes for viral vectors, with increasing support from myriad studies [61,92,93,94]. Their ability to be administered multiple times with negligible immune response from the host, coupled with their targetability, stability during storage, and ease of large-scale production, are key advantages driving their ongoing development [95]. There are various types of non-viral nanoparticle-based gene delivery systems (Figure 3) briefly discussed in this section.

### 8.1. Liposomes

Liposomes are nanoparticles composed of a phospholipid bilayer resembling a cellular membrane. Depending on their dimensions and layered structure, they can be categorized into three distinct types: multilamellar vesicles, small unilamellar vesicles, and large unilamellar vesicles. These lipid nanocarriers find wide applications in drug delivery systems due to their favorable size, biocompatibility, natural degradation, low toxicity, and ability to encapsulate drugs with various properties (hydrophilic, lipophilic, or amphiphilic) [96,97]. Liposomes show promise in retinal gene therapy for delivering nucleic acids. They offer targeted delivery and molecule encapsulation benefits. Researchers have created effective complexes containing cationic lipids, neutral lipids, cholesterol, protamine, and cell-penetrating peptides to achieve efficient and sustained gene expression in the retina [98]. In animal models, tiny liposome-based carriers delivered DNA plasmids, expressing genes, and partially curing diseases. By using cell-specific promoters, they improved gene expression in specific retinal cell types [99,100]. Cationic liposomes, made of positively charged compounds and neutral lipids, are vital for gene therapy delivery. Their positive charge helps form stable complexes with negatively charged genes, leading to longer circulation time and improved transfection efficiency [101]. Researchers have investigated novel liposomal non-viral vectors like exosome–liposome hybrids [99]. New gene therapy designs enhance drug delivery efficiency. Liposomes carrying CRISPR/Cas9 show promising results, with better-targeted gene knockout and lower toxicity than free CRISPR/Cas9. This advancement holds potential for disease modeling and treatment [102].

Masuda et al. tried three different kinds of cationic liposomal systems (based on TMAG, DDAB, and DC-cholesterol) to deliver pDNA encoding for the beta-galactosidase gene in ocular regions. All three systems were able to transfect various retinal layers except the photoreceptor cell [103]. Bochot et al. proposed a liposome system to encapsulate pdT16 nucleotide and observed a good release in poloxamer-containing media [104]. Kachi et al. evaluated the safety and efficacy of commercially available cationic liposomal formulations–lipofectamine 2000 and NeuroPorter. Again, both therapies were able to transfect retinal cell layers with the subretinal injections achieving transfection into deeper regions (compared to intravitreal injections) [105].

These promising effects offer an essential platform for nucleic acid delivery in retinal gene therapy. They provide efficient and targeted delivery, and their versatility makes them valuable in ophthalmic drug delivery.

### 8.2. Solid Lipid Nanoparticles (SLNs)

SLNs consist of a solid lipid core surrounded by a layer of surfactants in water. They should be smaller than 500 nm in diameter, with an ideal size of 120 nm or less for in vivo use [106,107]. SLNs offer several advantageous characteristics, including controlled and targeted drug release, improved stability of pharmaceuticals, and higher drug content compared to other carriers. They can carry both lipophilic and hydrophilic drugs, and due to most lipids being biodegradable, they exhibit excellent biocompatibility. SLNs utilize water-based technology, avoiding the need for organic solvents. They are also easy to scale up and sterilize, more cost-effective than polymeric or surfactant-based carriers, and simpler to validate for regulatory approval [108]. Cationic SLNs have emerged as a popular choice for gene delivery due to their ability to interact electrostatically with DNA, forming complexes known as lipoplexes. These lipoplexes serve as protective structures for DNA and facilitate targeted delivery to specific cells. Several studies have demonstrated the potential of cationic SLNs in gene delivery. The formation of DNA plasmid complexes with cationic solid lipid nanoparticles (SLNs) was successfully demonstrated [109]. A formulation called siRNA-PEG/SLN was developed, which showed the ability to cross the blood–brain barrier and target tumor sites without causing apparent systemic toxicity [110]. In another study, researchers employed cationically modified SLNs as carriers for RNA and examined their suitability as a non-viral vehicle for gene delivery [111].

As for testing on ocular tissues, dextran and protamine-based SLNs have been used to upregulate retinoschisin and EGFP in ARPE-19 cells, implicating usefulness for gene delivery for the retinal degenerative conditions [112]. Although some other studies have shown that SLNs are liable to intracellular trafficking and lysosomal binding in RPE cells, more research on strategies like surface functionalization should be conducted to overcome this demerit [113].

Overall, solid lipid nanoparticles (SLNs) have emerged as promising gene delivery systems, offering advantages such as small size, controlled drug release, and biocompatibility. Their cationic counterparts have demonstrated the potential for effective gene delivery, opening exciting possibilities for targeted therapies in various applications, including ocular and brain-related disorders.

### 8.3. Micelles

Micelles are self-assembled monolayered spheres formed when amphiphilic molecules are progressively added to a solution and surpass their critical micellar concentration (CMC) [114]. Micelles are considered advantageous for gene therapy due to their low size (typically under 100 nm), simplicity of synthesis, higher drug loading capacity owing to the presence of monolayer, size, and higher flexibility in optimization of formulation features via the introduction of surface modifications or use of block copolymers, etc. [15,115,116]. Gene therapy research has seen significant advancements with the use of micellar systems as non-viral vectors. Initial experiments demonstrated this potential by using a polymeric micelle complex to enhance the expression of the lacZ gene in the ocular tissues of mice and rabbits [117]. Subsequent research utilized the same block copolymer system, specifically PEO-PPO-PEO, for the delivery of genes like keratin 12 (K12) and keratocan into corneal tissues [118]. Additionally, fluorescence-labelled polyion micelles, loaded with dendrimer porphyrin, were employed in the treatment of choroidal neovascularization-induced injuries in rats, showcasing their applicability in ophthalmic conditions [119]. Furthermore, a micelle composed of Poly(ethylene oxide)–poly(propylene oxide)–poly(ethylene oxide) (PEO-PPO-PEO) was used to encapsulate and deliver the bcl-XL gene, an anti-apoptotic gene, to prevent corneal apoptosis [120].

### 8.4. Dendrimers

Dendrimers belong to a specialized category of dendritic polymers characterized by a meticulously structured design composed of branched units known as “branch cell monomers” [121,122,123,124,125]. They are hyperbranched, three-dimensional molecules with a unique architecture comprising a central core, numerous branching layers, and surface functional groups [121]. Cationic dendrimers, distinguished by their precise number of surface amine groups, are capable of compacting nucleic acids into minuscule nanoparticles via ionic interactions, thereby safeguarding them against enzymatic destruction [125,126,127,128,129,130,131,132]. In ocular delivery, lipophilic amino acid dendrimers and polyamidoamine dendrimers were successfully used [133]. Additionally, dendrimers have shown efficacy in reducing scar tissue formation post-glaucoma surgery in rabbits, exemplifying their role in minimizing inflammatory responses [134]. Liao et al. were able to transfect mice RGC cells with a PEI-based dendrimer system encapsulating p-DNA with shRNA genes to knock down melanopsin [135].

These findings suggest dendrimers’ versatility in gene delivery, making them a valuable tool in medical research and therapy.

### 8.5. Polymersomes

Polymersomes (Ps) are a unique class of artificial vesicles crafted from synthetic amphiphilic block copolymers [136]. These vesicles typically manifest as hollow spheres, encompassing an aqueous solution within their core, which is encased by a bilayer membrane. This membrane is distinctively structured, comprising hydrated hydrophilic coronas on both its internal and external surfaces, flanking a hydrophobic middle section. This design effectively isolates and safeguards the fluidic core from the external medium [137]. The aqueous core of these polymersomes is adept at encapsulating therapeutic molecules such as drugs, enzymes, other proteins and peptides, and DNA and RNA fragments [138,139,140,141,142,143]. A key feature of polymersomes is their stimuli-responsive drug release capability, which allows for them to modify their physical and chemical properties in response to various environmental stimuli, including pH, temperature, redox conditions, light, magnetic fields, ionic strength, or concentration [144]. Furthermore, polymersomes offer increased circulation times, decreased macromolecule degradation, and reduced immune responses, which are critical factors in the successful delivery of therapeutic agents like nucleic acids [145]. Glycol chitosan NPs containing pDNA were able to transfect RPE cells in wild-type albino mice, delivered via subretinal injection [146]. Block copolymer systems of poly-L-lysine and PEG have been used to deliver genes to photoreceptor, RPE, and retinal ganglion cells [13,147,148,149,150].

These properties collectively enhance the delivery efficiency and reduce the cytotoxic side effects associated with nucleic acid therapies.

### 8.6. Niosomes

Niosomes are regarded as promising alternatives for developing novel formulations for gene and oligonucleotide transfection. They are non-viral vectors akin to liposomes, but they replace phospholipids with non-ionic surfactants [151]. In addition to non-ionic surfactants, cationic lipids are incorporated into niosomes for gene delivery, facilitating the complexation of nucleic acids with cationic niosomes through simple electrostatic interactions [152]. Recently, a group of researchers created niosomes containing BSA, showcasing their ability to control the release of the protein [153]. Additionally, a team formulated methylene blue (MB)-loaded niosomes, underscoring their high encapsulation efficiency and potential for wound healing [154]. Retinal gene delivery was enhanced by incorporating lycopene into cationic niosomes. They effectively targeted the inner layers of the retina and transfected the outer segments through subretinal and intravitreal injections in rats [155]. A study evaluated chloroquine diphosphate in cationic niosomes for rat retina transfection, finding enhanced efficiencies with chloroquine-enhanced nioplexes [156]. Another study investigated niosomes with different surfactants, revealing that those with polysorbate 20 were most effective in transfecting retinal cells, suggesting their potential in non-viral gene therapy [157,158].

### 8.7. Inorganic Nanocarriers

Inorganic nanocarriers show promise with easy preparation, storage, large surface area, stability, and customizable features. However, their biosafety remains controversial, requiring further research. Surface modification can enhance transfection efficiency and reduce toxicity [101]. They can be divided into several categories: metallic nanoparticles, metal oxide nanoparticles, nanoparticles containing doped metals or metal oxides, and metal sulfide and metal–organic frameworks. Metallic nanoparticles such as silver (Ag), gold (Au), copper (Cu), magnesium (Mg), titanium (Ti), platinum (Pt), zinc (Zn), and iron (Fe) nanoparticles have been explored in various fields of study and have proven to be effective and stable platforms for drug delivery [97]. While silver nanoparticles have valuable antifungal, antioxidant, anti-angiogenic, and anti-inflammatory properties, their use in ocular drug delivery is hindered by documented toxicity [97,159]. Gold nanoparticles are advantageous over other nanoparticles due to their chemical stability, biocompatibility, surface functionalization, and unique surface characteristics [97,160]. In inherited retinal dystrophies, researchers have explored them as a safer and more effective option than viral methods for delivering genes to RPE cells. In a study by Trigueros et al., the efficiency of transfection AuNPs loaded with p-DNA on an ARPE-19 cell line was evaluated by fluorescent tagging with EGFP plasmid and fluorescence microscopy along with immunohistochemical analysis with rabbit anti-FGFP antibodies, the 40 nm plasmid DNA-coated AuNPs were able to transfect differentiated ARPE-19 cells, with an efficiency comparable to that of liposomes [97,161].

Inorganic nanocarriers offer a wide range of benefits for drug delivery, but their biosafety concerns necessitate further investigation. Surface modification strategies, including the use of metallic nanoparticles like gold, hold promise for enhancing their performance and safety in various applications. Some of the non-viral gene delivery systems utilized for gene delivery are presented in the Table 1.

## 9. Limitations of Nanocarrier Systems for Ocular Gene Therapy and Their Comparative Evaluation

Overall, all nanoparticle-based (non-viral) gene delivery systems face the common problem of having transient gene expression compared to their viral vector counterparts. Their systemic accumulation (especially in the case of gold NPs) can cause toxicity over time, rendering them unsuitable for long-term use [170,177]. Stability issues arise for micelles, SLNs, polymersomes, and niosomes, which limits their scalability and applicability to clinical therapy [116,167,174]. In case of niosomes, surface modification with cationic surfactants is required to increase binding/loading of negatively charged nucleic acids; however, the surface modification increases the chances of toxicity [176].

Immunogenicity is another commonly shared concern. Many nanodelivery vectors after nucleic acid release can degrade to toxic constituents (charged lipids/metal ROS) or are rapidly uptaken by the RES (extracellularly) or endosomal systems (intracellularly), leading to the nucleic acid cargo getting broken down before it can express the therapeutic gene. This is why, there are currently no large-scale clinical trials ongoing for testing non-viral vector-based ocular gene delivery [116,166,171,173,175]. Two nanodelivery systems, liposomes and micelles, show promise in overcoming many of these limitations.

Liposomes can be surface-modified with PEG to increase permeation via ocular barriers as well as avoid antigenic detection/ubiquitination. Liposomes even without surface modification have good permeation, and with surface modifications can achieve targeted delivery as well as controlled release kinetics. Liposomal delivery of aptamers via intravitreal administrations appears to be the closest non-viral gene delivery approach to see the light of clinical testing [26,165].

Micelles made from diblock, triblock, and graft copolymers can have PEG as one of the hydrophilic blocks to increase barrier permeations as well as impart stealth properties. They have higher loading capacity than liposomes and can be surface-modified to achieve niche delivery objectives as well, making them the second most prospective candidate for ocular gene delivery [115,178].

## 10. Current Progress in Clinical Trials of Gene Therapy for Retinal Disorders

Considering that the earliest therapeutic approaches for gene therapy were constructed around viral vectors, it is no surprise that a larger proportion of viral vectors are currently under clinical screening. This is because converting nanomedicine to clinical therapy is tricky due to its cytotoxicity. Table 2 describes a list of some of the viral vectors for ocular gene delivery currently under clinical trial.

Most of the mentioned therapies were demonstrated to have good tolerability. Viral vectors have higher transfection efficiency; however, they also have considerable immunogenicity, with multiple trials reporting mild ocular inflammation as a common adverse effects (AE). Some trials even resort to keeping oral/topical anti-inflammatory medication as a supplemental therapy/prophylactic measure for such events.

Ocular discomfort, hypotony, conjunctival hemorrhage, infections to ocular tissues, and even extraocular AEs may occur. Another factor that makes viral gene delivery less desirable is the need for surgical interventions for certain administration routes (e.g., intravitreal injections), this creates a lot of surgery-related AEs.

Efficacy-wise, many of the listed therapies were able to improve patient outcomes, which was quantified based on parameters like best correlated visual activity BCVA, perimetry, low- luminescence visual activity (LLVA), etc., measured using standardized scales like logarithm of minimum angle of resolution (LogMAR), visual activity Score (VAS), Snellen chart, early treatment diabetic retinopathy study(ETDRS), etc.

## 11. Conclusions

In this review, we explored the evolving landscape of non-viral vectors, with a specific focus on the challenges and advancements in treating inherited retinal disorders. We begin with an overview of eye anatomy and the barriers within the ocular environment, followed by a discussion on common retinal disorders, current therapies, and their limitations. Subsequently, we examine various nanoparticle-based gene therapy delivery systems, highlighting their potential in overcoming long-standing challenges. The review also includes a summary of the current progress in clinical trials of gene therapy.

The selection of gene carriers—viral or non-viral—is pivotal in gene therapy. The primary challenge remains the delivery of nucleic acids that represents a significant challenge in the field of drug delivery. The development of gene therapy has been enhanced by emerging biological technologies. However, the genetic diversity of diseases, the complexity of their pathogenesis, and individual variations present considerable challenges in clinical trials and treatments. The use of viral vectors for carrying nucleic acids, with their efficiency, yet associated risks, along with the high costs of research and development, are further obstacles. So, advances are anticipated in reducing immunogenicity, increasing target specificity, and enhancing the transduction efficiency of vectors. Non-viral techniques, like lipid-based nanoparticles or polymer systems, may become key components of future vector designs. Ethical considerations are also crucial, especially considering the potential unforeseeable consequences of gene modifications. Future studies are expected to dive deeper into basic research for more effective targets, address delivery challenges, and optimize clinical trial protocols for more precise treatment outcomes.

Over the past few years, retinal gene therapy has made significant progress. Despite these advancements, there is still a pressing need to address the economic burden and enhance patient well-being. In conclusion, gene therapy holds immense promise in treating genetic diseases. With continued research and the exploration of new vectors, it has the potential to revolutionize future treatments significantly. Successes in retinal therapy, such as Luxturna, offer hope for more breakthroughs that can substantially improve patient quality of life.

## Figures and Tables

**Table 1:** Advantages of non-viral vector-based gene therapies.

Sr No.	Name of Non-Viral Gene Delivery System	Specific Advantages	Nucleic Acid Used for Delivery	Limitations
1.	Liposomes	Biocompatibility, natural degradation, enhanced permeability by annexin-5-mediated endocytosis, and ease of introducing surface functionalizing for active targeting.	siRNA [162,163] and pDNA [164]	Immunogenicity, short residence time on eye surface [165]
2.	Solid lipid nanoparticles (SLNs)	Sustained drug release, high stability in vivo, and cost-effective scale-up.	pDNA, siRNA and mRNA [111]	Initial burst release, low drug loading efficiency, possibility of crystallization during storage [166,167]
3.	Micelles	Large gene loading capacity, simplicity of synthesis.	pDNA [115,117,118,119,120]	High tear clearance, swift disintegration upon dilution in body fluids and immunogenicity [116]
4.	Dendrimers	Versatility in synthesis as per need, high loading capacity and customizable synthesis (based on number of generations).	Antisense oligonucleotides [168], pDNA [126,127], mRNA, and siRNA [168,169]	Intrinsic toxicity of traditional dendrimer ingredients, complexity of synthesis [170,171]
5.	Polymersomes	Stimuli responsive drug release and reduced cytotoxic effects.	pDNA [172,173]	Low drug loading efficiency, irregular shape (unruly self-assembly), possibility of aggregation [174,175]
6.	Niosomes	High stability, long circulation half-life, and release kinetics similar to liposomes.	pDNA [155,156,157]	Low entrapment efficiency (compared to liposomes), possibility of cargo leakage, high possibility of fusion events [176]
7.	Inorganic nanocarriers (AuNPs)	Large surface area, ease of surface modification, and customizable features.	pDNA [160,161]	Clearance issues, toxicity at higher concentrations, and non-specific targeting [177]

**Table 2:** Viral vector-based ocular gene therapies in clinical trials.

Condition	Description	Vector	Clinical Phase	NCT Numbers	Safety Results
Achromatopsia	Non-randomized, open-label CLARITY clinical trial with treatment being recombinant adeno-associated virus vector expressing CNGB3 administered via subretinal injection route.	rAAV2	1/2	NCT02599922	No official data posted (as of January 2025).
	Non-randomized, open-label interventional clinical trial with treatment AGTC-402 administered to one eye by subretinal injection.	rAAV2	1/2	NCT02935517	Phase 1 study showcased a good safety profile [179].
Age-related macular degeneration	Non-randomized, interventional multicenter trial with treatment GT005 administered as a single subretinal injection (3 doses) in genetically defined subjects with macular atrophy.	rAAV	1/2	NCT03846193	In phase I study, mild ocular inflammation events were observed in some patients. Study has been terminated [180].
	Open-label prospective multicenter trial with treatment ixo-vec via intravitreal route.	AAV.7m8	2	NCT05536973	Intraocular inflammation was observed in some patients but were manageable with topical corticosteroids [181].
	Randomized, partially masked, interventional parallel assignment trial with RGX-314 gene therapy given via an outpatient surgical procedure.	AAV8	2/3	NCT04704921	Results not posted (as of January 2025).
	Non-randomized, open label interventional clinical trial with sequential assignment. Treatment RGX-314 administered via subretinal delivery (2 doses)			NCT04832724	Trial is completed but results are not officially posted (as of January 2025).
	Randomized, partially masked interventional study with parallel assignment. Treatment RGX-314 given as single subretinal injection with in two different doses in different treatment arms.			NCT05407636	No data posted yet (in recruiting stage as of January 2025).
	Randomized, open-label, controlled masked expansion clinical trial with sequential assignment. Treatment 4D-150 given via intravitreal injection in multiple dose groups.	AAV	3	NCT05197270	No official data posted yet (in recruiting stage as of January 2025).
Choroideremia	Non-randomized, long-term safety and efficacy follow-up study for AAV2-REP1 treatment for patients with chloridemia and AAV8-RPGR treatment for X-linked retinitis pigmentosa, both given via subretinal injections.	AAV2	3	NCT03584165	No data posted yet (in recruiting via invitation as of January 2025). As predecessor study, reported inflammation and surgery related adverse events (NCT02407678).
Diabetic macular edema	Randomized, double-masked, multicenter, controlled interventional clinical trial with parallel assignment. Treatment ADVM-022 given by one-time intravitreal injection.	AAV.7m8	2	NCT04418427	No official data posted (as of January 2025).
Diabetic retinopathy	Randomized, partially masked dose-escalation, observational controlled clinical trial with parallel assignment. Treatment RGX-314 given via single suprachoroidal space (SCS) injections.	AAV8	2	NCT04567550	No data posted (recruiting as of January 2025).
	Long term follow-up prospective observational study (no intervention).			NCT05296447	No available data (enrolling as of January 2025).
Autosomal recessive Leber’s congenital amaurosis	Non-randomized, open label-controlled dose-escalation interventional study. Treatment ATSN-101 administered as unilateral subretinal injection.	AAV5	1/2	NCT03920007	AEs ranging from ocular discomfort and conjunctival hemorrhage to infections in various ocular tissues; however, the product was overall considered tolerable [182].
Leber’s congenital amaurosis (LCA)	Non-randomized, open-label single ascending dose interventional study with sequential assignment. Treatment EDIT-101 administered via subretinal injection to multiple dose groups.	AAV5	1/2	NCT03872479	No SAEs or dose-limiting toxicity events observed [183].
	Long term follow-up prospective observational study (no intervention).	AAV2/5	Follow-up	NCT02946879	No available data despite study completion.
	Follow-on dose escalation and safety intervention study with multiple dosing groups. Treatment was given as a single-dose unilateral subretinal injection.	AAV2	1/2; 3; follow-up	NCT01208389	No adverse events related to AAV reported [184].
	Post-authorization long term (5-year), multicenter, longitudinal observational patient registry with cohorts. Original treatment was a vector, AAV2-hRPE65v2, given subretinally.			NCT03597399	No results posted (as of January 2025).
	Randomized, open-label interventional study at 2 sites. Treatment AAV2-hRPE65v2 (voretigene neparvovec-rzyl) was administered subrationally.			NCT00999609	No treatment-related SAEs or serious immune responses observed [185].
	Long-term follow-up prospective observational study in cohorts previously subretinally administered with (AAV2-hRPE65v2, voretigene neparvovec-rzyl).			NCT03602820	No data posted (expected to be completed by 2030).
Leber hereditary optic neuropathy	Prospective open-label proof of concept study conducted on 5 participants. Vector given via intravitreal injection.	AAV2	1	NCT02161380	No serious safety problems observed [186].
	Randomized, double-masked multicenter placebo-controlled interventional study. Treatment GS010 was administered via intravitreal injection.	rAAV2/2	3	NCT03293524	No official data available.
Retinitis pigmentosa	Non-randomized, open-label dose-escalation clinical trial with sequential assignment. Treatment GS030 administered as a single intravitreal injection.	AAV2	1/2	NCT03326336	No official data posted. Study yet to be completed (as of January 2025).
	Non-randomized, open-label dose-escalation clinical trial with sequential assignment. Treatment BS01 was given via intravitreal injection.	rAAV	1/2	NCT04278131	No official data available (as of January 2025).
	Non-randomized, open-label monocentric interventional study with sequential arrangement. Treatment HORA-PDE6B was given as a unilateral subretinal injection.	AAV2/5	1/2	NCT03328130	No official data posted (as of January 2025).
	Randomized, triple-masked, sham-controlled interventional study with sequential assignment. Treatment MCO-010 (optogenetic therapy) was given as an intravitreal injection.	AAV2/5	2	NCT04945772	As per the first 52-week data on 16 patients, no treatment-related SAEs were observed [187].
	Non-randomized, open-label clinical trial with sequential assignment. Treatment OCU400 given via single-dose subretinal injection in multiple dosing groups.	AAV5	1/2	NCT05203939	No official data posted (as of January 2025).
	Open-label interventional study with sequential assignment. Treatment rAAV.hPDE6A given via subretinal injection.	rAAV8	1/2	NCT04611503	Preliminary results reported that the treatment is well tolerated; however, some patients experienced vision loss [188].
	Non-randomized, open-label, quadruple-masked dose-escalation intervention study with single group assignment. Treatment rAAV2tYF-GRK1-RPGR administered subrationally.	rAAV2	1/2	NCT03316560	No published data (as of January 2025).
	Randomized, partially masked controlled interventional study with parallel assignment. Treatment AAV5-hRKp.RPGR given as a bilateral subretinal injection.	AAV5	3	NCT04671433	Study is completed but results are not yet published (as of January 2025).
X-linked retinoschisis	Non-randomized, open-label, multicenter dose-escalation intervention study. Treatment rAAV2tYF-CB-hRS1 vector given via intravitreal delivery.	rAAV2	1/2	NCT02416622	The gene augmentation therapy was generally safe and well tolerated [189].
	Non-randomized, prospective monocentric interventional study with single group assignment. Treatment AAV-RS1 vector was administered as intravitreal injection.	AAV8	1/2	NCT02317887	Mild ocular inflammation events that could be treated with corticosteroids/oral prednisone. One patient experienced a non-treatment-related SAE [190].
Stargardt disease	Open-label, multicenter interventional study with single group assignment. Treatment vMCO-010 (optogenetic therapy) given as single intravitreal injection.	AAV2	2	NCT05417126	The trial is completed, but official study data are not yet published (as of January 2025) [191].

## Abbreviations

AAV	adeno-associated viruses
AAV2-REP1	adeno-associated viral vector 2-Rab escort protein 1
ABCA4	ATP-binding cassette subfamily A member 4
ADRP	autosomal dominant retinitis pigmentosa
AONs	antisense oligonucleotides
ARRP	autosomal recessive retinitis pigmentosa
BAB	blood–aqueous barrier
BCVA	best-correlated visual activity
BD	best disease BD
BRB	blood–retina barrier
CERKL	ceramide kinase-like
CHM	choroideremia
CMC	critical micellar concentration
CNGB3	cyclic nucleotide-gated channel subunit beta 3
CRB1	crumbs family member 1
DDAB	dimethyl dioctadecyl ammonium bromide
DSBs	double-stranded breaks
EGFP	enhanced green fluorescent protein
ERG	electroretinogram
ETDRS	early treatment diabetic retinopathy study
EYS	eyes shut homolog
GUCA1B	guanylate cyclase activator 1B
GUCY2D	guanylate cyclase 2D
hESCs	human embryonic stem cells
hhRz	hammerhead ribozymes
hiPSCs	human-induced pluripotent stem cells
IMPDH1	inosine monophosphate dehydrogenase 1
IRDs	inherited retinal disorders
KLHL7	Kelch-like family member 7
LCA	Leber congenital amaurosis
LCA1	Leber congenital amaurosis 1
LLVA	low-luminescence visual activity
LogMAR	logarithm of the minimum angle of resolution
NR2E3	nuclear receptor subfamily 2 group E member 3
PDE6A	phosphodiesterase 6A
PDE6B	phosphodiesterase 6B
pDNA	plasmid DNA
PEO-PPO-PEO	poly(ethylene oxide)–poly(propylene oxide)–poly(ethylene oxide)
PLGA	poly(lactic-*co*-glycolic) acid
PRPF8	pre-mRNA processing factor 8
PRPH2	peripherin 2
rAAV	recombinant adeno-associated viruses
RHO	rhodopsin
RP	retinitis pigmentosa
RP1	retinitis pigmentosa 1
RPE	retinal pigment epithelium
RPE65	retinal pigment epithelium 65
RPGR	retinitis pigmentosa GTPase regulator
SAG	S-antigen visual arrestin
SEMA4A	Semaphorin 4a
siRNA	short interference RNA
SLNs	solid lipid nanoparticles
SNRNP200	small nuclear ribonucleoprotein 200
STGD	Stargardt disease
TALENs	transcription activator-like effector nucleases
TMAG	N-(α-trimethylammonioacetyl)-distearoyl-D-glutamate chloride
USH2A	Usher syndrome 2A
XLRP	X-linked retinitis pigmentosa.
ZFNs	zinc finger nucleases
